# The Effects of Vestibular Rehabilitation on Poststroke Fatigue: A Randomized Controlled Trial Study

**DOI:** 10.1155/2022/3155437

**Published:** 2022-08-31

**Authors:** Amin Ghaffari, Bahador Asadi, Armin Zareian, Malahat Akbarfahimi, Gholam Reza Raissi, Fahimeh Fathali Lavasani

**Affiliations:** ^1^Department of Neurology, Faculty of Medicine, AJA University of Medical Sciences, Tehran, Iran; ^2^Rehabilitation Research Center, Department of Occupational Therapy, School of Rehabilitation Sciences, Iran University of Medical Sciences, Tehran, Iran; ^3^Public Health Department, Health in Disaster & Emergencies Department, Nursing Faculty, AJA University of Medical Sciences, Tehran, Iran; ^4^Neuromusculoskeletal Research Center, Department of Physical Medicine and Rehabilitation, School of Medicine, Iran University of Medical Sciences, Tehran, Iran; ^5^Clinical Psychology Department, Behavioral Sciences & Mental Health School (Tehran Psychiatry of Institute), Iran University of Medical Sciences, Tehran, Iran

## Abstract

**Background:**

A major complication caused by stroke is poststroke fatigue (PSF), and by causing limitations in doing activities of daily living (ADL), it can lower the quality of life.

**Objective:**

The present study is an attempt to examine the effects of vestibular rehabilitation on BADL (Basic Activities of Daily Living), fatigue, depression, and Lawton Instrumental Activities of Daily Living (IADL) in patients with stroke.

**Method:**

Patients with a history of stroke took part voluntarily in a single-blind clinical trial. The participants were allocated to control and experimental groups randomly. The experimental group attended 24 sessions of vestibular rehabilitation protocol, while the control group received the standard rehabilitation (including three sessions per week each for around 60 min). To measure fatigue, the Fatigue Impact Scale (FIS) and the Fatigue Assessment Scale (FAS) were used. Depression, BADL, and IADL were measured using the Beck Depression Inventory-II (BDI-II), Barthel Index (BI), and Lawton Instrumental Activities of Daily Living, respectively. All changes were measured from the baseline after the intervention.

**Results:**

Significant improvement was found in the experimental group compared to the control group (*p* < 0.05) in FIS (physical, cognition, and social subscales), FAS, BDI-II, BADL, and IADL. Moreover, the results showed small to medium and large effect sizes for the physical subscale of FIS and FAS scores based on Cohen's *d*, respectively; however, no significant difference was found in terms of cognition and social subscales of FIS, BDI-II, BADL, and IADL scores.

**Conclusion:**

It is possible to improve fatigue, depression, and independence in BADL and IADL using vestibular rehabilitation. Thus, it is an effective intervention in case of stroke, which is also well tolerated.

## 1. Introduction

Fatigue is a very common poststroke complication, with a prevalence of 16-74% [1, 2], which is considered one of the symptoms of poststroke depression (PSD) [3]. However, the fact that patients without depression frequently complain of fatigue necessitates a study of “poststroke fatigue” (PSF) as a specific syndrome [4].

The syndrome manifests itself as a sense of helplessness, lack of energy, and excessive burnout. The syndrome is different from normal and nonpathological fatigue, which is mostly because of the side effects of drugs, heavy exercise, or diseases [4, 5]. This type of fatigue is a chronic condition that may be the only symptom in stroke patients, even with an excellent neurologic recovery, and may persist for many years [1, 6].

The PSF is associated with functional deficits and participation restrictions in the activities of daily living (ADL) and leads to a lower quality of life [7, 8]. In addition, the occurrence of fatigue during daily occupations is associated with psychological disorders such as depression [9]. Therefore, PSF is seen as a major complaint that needs more efficient management in patients with stroke.

Several studies have been carried out to elaborate on the mechanisms of PSF; however, the etiology is still unclear [10, 11]. One reason for this is the several factors in PSF and that studies can only work on some of these factors to treat, prevent, or manage PSF [12–14]. Studies on the sensory integration theory in patients with stroke have shown deficits in sensory registration and modulation leading to restricting their participation in daily occupations [15–17]. Disruption of the central sensory-motor integration such as visual, somatosensory, and vestibular systems leads to an unbalanced use of these senses by patients with stroke [18]. This lack of integrated use can lead to inefficient postural control that may be due to fatigue [16]. Visual, somatosensory, and vestibular systems are the key elements of the central sensory-motor integration, and it can be triggered positively through vestibular rehabilitation [19]. As a training program, vestibular rehabilitation contributes to sensory integration through modulating self-awareness, body, space, and spatial navigation and reflex generation for oculomotor and posture control by adaptation compensatory mechanisms through repeating tasks [20].

Recent studies on stroke patients receiving vestibular rehabilitation have shown an improvement in gait performance, balance, and self-perceived health [21–24]. This result is also supported in stroke patients, so that neurophysiological findings show that the vestibular cortical network is strongly related to the rest of sensory and motor signals, memory, attention, social cognition, and mental imagery [25–27]. Moreover, long-term decrease in hemispatial caused by subliminal galvanic vestibular stimulation improves anticipatory postural adjustment (APA) and vertical perception, which results in a quick and efficient performance with less energy expenditure in stroke patients [28, 29]. In spite of all evidence, there is no study on using VR programs to lower fatigue in stroke patients.

With this introduction, the present work is an attempt to examine the effects of vestibular rehabilitation training on the fatigue in stroke patients. The main hypothesis of the study is that a neurorehabilitation training such as vestibular rehabilitation can attenuate fatigue as the primary outcome and depression severity and independence in basic and instrumental ADL as secondary outcomes in patients with stroke.

## 2. Methods

The study was carried out as an interventional work based on a single-blind clinical design. The participants were selected through convenience sampling among stroke patients who were army retirees and referred to Golestan and Imam Reza Hospitals affiliated with AJA University of Medical Sciences. After a pilot study and based on the exclusion and inclusion measures, 32 participants were selected and allocated to control and experimental groups randomly (16 patients in each group). The interventions were performed by a senior occupational therapist who was blind to the grouping. This study was conducted between January and May 2022.

### 2.1. Standard Protocol Approvals

The selected participants signed an informed letter of consent, and the Ethical Committee of AJA University of Medical Sciences (IR.AJAUMS.REC.1400.245) and the Iranian Registry Center of the Clinical Trials (IRCT20090904002415N3) approved the study.

### 2.2. Sample Size and Randomization

According to the sample size estimation procedure, a pilot study was performed with 6 subjects. Based on the results, the mean ± SD of the Fatigue Impact Scale was 87.16 and 8.03, respectively. Using the G∗Power 3.1 software in terms of 95% confidence level and power 93% by considering *α* = 0.05 and *β* = 0.95 and assuming two-tailed, the sample size was equal to 34 patients. Two patients were excluded during the study (attrition rate = 5.88%), and finally, the study was performed with 32 stroke patients. Participants were randomly allocated (allocation ratio 1 : 1) to one of the two groups (experimental group = 16 and control group = 16) with permuted block randomization method by the research coordinator.

### 2.3. Participants

**Figure 1:**
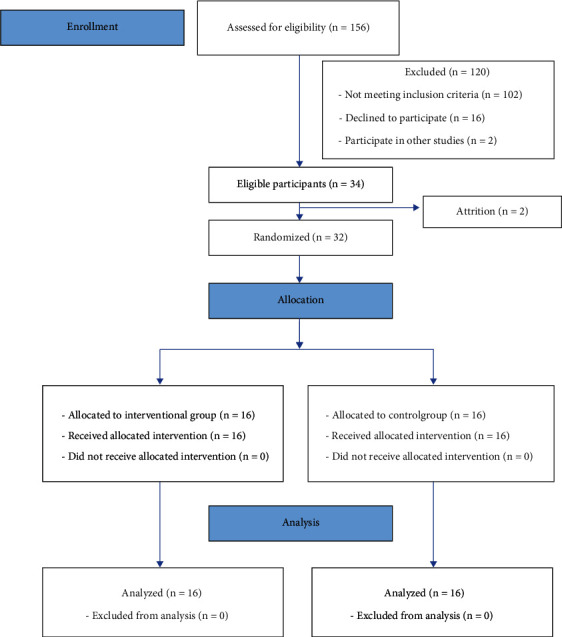
Flowchart of the study.

The participants were selected based on the following inclusion criteria: stroke with unilateral hemiplegia in the past 6 to 36 months ago, able to walk with no need to any device or continuous physical support for the body weight or to keep the balance (Functional Ambulation Classification ≥ 3), and Fatigue Assessment Scale (FAS) score ≥ 24 [30]. There were also exclusion criteria, namely, cognitive problems that might affect one's ability to comprehend instructions (Mini-Mental State Examination < 24) [31], severe aphasia, severe unilateral spatial neglect, and neurological and orthopedic comorbidities like significant osteoarthritis, particularly in the lower limbs. Additionally, the participants reluctant to cooperate during the study or those who had a change in their medication protocols were excluded (Figure 1).

### 2.4. Assessment Tools

The Fatigue Impact Scale (FIS) questionnaire was used to evaluate the various dimensions of fatigue [32]. This instrument contains 40 items that assess functional limitations due to fatigue in three fields of everyday life, including cognitive functioning (10 items), physical functioning (10 items), and psychosocial functioning (20 items). The questions are designed based on a Likert's five-point scale (4 = extreme problem,…, 0 = no problem). The total score of the tool ranges from 0 to 160, and the total score of the domains ranges from 0 to 80 for psychosocial functioning and from 0 to 40 for physical and cognitive functioning. Higher scores indicate greater limitations in functioning [33]. The Persian version of the FIS (FIS-P) has been psychometrically evaluated in patients with stroke. The intraclass correlation (ICC) values for interrater reliability on the cognitive subscale, social subscale, physical subscale, and total score were 0.86, 0.95, 0.89, and 0.98, respectively. In addition, the test-retest reliability values were equal to 0.78, 0.92, 0.86, and 0.93, respectively. The Cronbach's alpha of the FIS-P was equal to 0.95, i.e., a high reliability [34].

The Fatigue Assessment Scale (FAS) questionnaire was used to evaluate symptoms of fatigue [35]. This self-report questionnaire has 10 items designed based on Likert's 5-point scale (1 = never,…, 5 = always). The total FAS score ranges from 10 to 50, so that the higher the score, the higher the fatigue. Despite other similar measures (such as the Fatigue Impact Scale), the FAS approaches fatigue as a unidimensional construct without diverse factors. The cut-off point for fatigue in stroke patients was set at 24 based on the FAS [30]. As to internal consistency of the Persian version, Cronbach's alpha coefficient for physical and mental fatigue was 0.945 and 0.896, respectively [36, 37].

Beck Depression Inventory-II (BDI-II) was used to examine depression severity [38]. The BDI-II includes cognitive, motivational, emotional, and physiological dimensions. This scale includes 21 items about the respondent's feelings in diverse situations over the past week, and the items are scored from 0 to 3. The BDI-II demonstrated a significant test-retest reliability (*r* = 0.64) and a positive internal consistency (alpha = 0.92) in the Iranian population [39].

To measure the performance of patients in terms of basic ADL (e.g., grooming, bowel and bladder function, feeding, toilet use, mobility, dressing, transfer, steps, and taking bath), Barthel Index (BI) was used. The tool score ranges from 0 (complete dependency) to 100 (completely independence) [40]. The reliability of the Persian version of BI is significant at 0.938, and it has a good validity in Iranian population [41].

Lawton Instrumental Activities of Daily Living Scale was used to examine activities like shopping, using mobile phone, housekeeping, food preparation, laundry, transportation, adhering to medication, and handling financial affairs. The tool score ranges from 0 for dependence and low function to 8 for independence and high functioning [42]. Studies have supported test-retest reliability of the Persian version of the Lawton IADL Scale (*r* = 0.96) in Iranian population [43].

### 2.5. Interventions

Vestibular rehabilitation practices were carried out by the participants in the experimental group for around 60 min at all sessions three days per week. The vestibular rehabilitation exercises consisted of workout on trampoline, firm surface, foam, and a balance board with eyes open and close; sideways, upward, and downward head motions; throw and catch ball with alterations in the center of gravity; walking while moving the head; and moving a ball in hands from side to side. Oculomotor exercises would be carried out for around 10 min every session [19, 44] (Appendix A). On the other hand, the control group in this study received 24 sessions of conventional rehabilitation interventions for about 60 minutes (three times a week) including stretching, strengthening, and range of motion exercises for the limbs and trunk based on the stroke rehabilitation guidelines [45]. The patients were allowed to rest during the training if they felt tired.

### 2.6. Statistical Analysis

Data analyses were done in SPSS (v20.0) (SPSS Inc., Chicago, IL, USA). The Mann–Whitney *U* test was used for BADL and IADL variables. In addition, FIS, FAS, and BDI-II were analyzed by independent sample *t*-tests. The total scores of FIS, FAS, BDI-II, BADL, and IADL were normally distributed. Wilcoxon signed rank test and paired *t*-tests were used for comparing the variables in both intervention and control groups (*p* = 0.05). The significance level was modified familywise for multiple comparisons using the error-rate method. Cohen's *d* was obtained and interpreted as 0.20 referring to a small effect, 0.50 referring to a medium effect, and 0.80 referring to a large effect [46].

## 3. Results

In total, 32 male patients completed the intervention programs. As listed in Table 1, the experimental group (mean age 58.27) and control group (mean age 61.61) had no significant difference in terms of demographic variables (age, educational level, marital status, blood pressure, diabetes, cardiac disease, affected limb, and time since stroke) and clinical characteristics (FIS, FAS, BDI-II, BADL, and IADL) of the participants at baseline (*p* > 0.05).

The results of paired *t*-tests in the experimental group revealed a significant decrease in both the total scores of FAS and all subscales (physical, cognition, and social) of FIS (*p* < 0.001); however, no difference was observed in the control group. In addition, the experimental group had a significant change in terms of BDI-II scores (*p* < 0.05), while there was no significant change in the control group (*p* > 0.05).

Furthermore, and compared to the control group, the results of the Wilcoxon signed rank test showed that there was a significant change in BADL and IADL scores in the experimental group (*p* < 0.05 and *p* > 0.05).

Independent sample *t*-tests revealed a significant difference in the physical subscale of FIS (*p* < 0.05, effect size = 0.38) and FAS (*p* < 0.05, effect size = 1.34) scores between the two groups after the treatments (Table 2).

## 4. Discussion

The effects of vestibular rehabilitation exercises on fatigue rate, depression severity, and independence in the instrumental and basic everyday activities in patients with stroke were examined.

The primary variable in this study was a change in fatigue, and it was improved in the experimental group by 8.61 and 5.22 in terms of FIS (total) and FAS compared to 0.28 and 0.27 in the control group (*p* < 0.001). Furthermore, the results indicated small to medium and large effect sizes of the vestibular rehabilitation program on the physical subscale of FIS and FAS scores between the two groups based on Cohen's *d* effect sizes, respectively. This is the first study on, as far as the authors know, the probable effects of vestibular rehabilitation on fatigue in patients with stroke. Thus, it is not possible for us to make a comparison with other studies; still, Abasi et al. and Hebert et al. examined the role of vestibular rehabilitation in fatigue in Parkinson's and MS patients, respectively [47, 48]. The purpose of this study was not to examine the underlying mechanism of fatigue improvement. The results give us a clearer picture of the neural adaptation after stroke [49]. In the case of damage to the central nervous system, sensory adaptation occurs, and this procedure creates a pattern of sensory dependence [25, 50]. It seems that vestibular exercises improve central nervous system adaptation and reduce the pattern of sensory dependence, thus reducing the patient's overreliance on the visual and somatosensory systems to make a more use of the vestibular system [29]. Therefore, with resensory integration, a balance is struck between the brain's capabilities leading to reduced fatigue and less energy expended on daily tasks. Electroneurophysiological evidence showed abnormal connectivity of supplementary motor area (SMA) and primary motor cortex (M1) in stroke patients [51]. The results indicate that by stimulating the supplementary motor area (SMA) using vestibular training in dynamic conditions with no visual differences, we may be able to reorganize SMA-to-M1 connectivity patterns along with generating a sensory-motor gain, which helps improving the anticipatory postural adjustment (APA) and achieving a higher performance in doing everyday activities [52]. These results are consistent with Tramontano et al. and Mitsutake et al. who showed that vestibular rehabilitation in patients with stroke had positive effects on patients' postural control and balance in walking [21, 23]. Probably, keeping the balance following vestibular therapy requires less energy, and this can improve poststroke physical fatigue. A high level of cognition and mild depression score of participating stroke patients might be one explanation for no difference between the groups in cognitive and social subscales of FIS. It seems that to improve all dimensions of fatigue, it is better to consider all aspects of occupational performance such as the person, environment, and activity components during rehabilitation interventions. In general, as PSF is a modifiable variable, rehabilitation professionals in rehabilitation centers need to manage poststroke fatigue in addition to providing necessary interventions to improve participation in various contexts.

The second variable of our study was the severity of depression. Changes in BDI-II scores for the experimental group were significant. These changes are consistent with the known association between depression and fatigue following stroke [53, 54]. It should be noted that in the comparison between the groups after treatment, no significant difference was found in depression. A small sample size might be one explanation for this. Other possible explanations might be the time since stroke onset along with cognition ability and their interaction with poststroke depression (PSD) in our study [55–57]. Additionally, it should be noted that the mean depression scores before the interventions were in the mild range and potentially affected our findings. Moreover, the vestibular rehabilitation protocol may need to be revised and seen in the form of purposeful and meaningful activities that creates more motivation in stroke patients [58].

The third major finding of this study was that the experimental group improved significantly in terms of independence in basic and instrumental ADL; however, the difference was not significant between the groups following the treatment. Our findings in the experimental group are consistent with Dai et al. who showed that vestibular exercise in unilateral neglect patients with the right hemispheric stroke improved ADL, neglect, and balance over time [59]. On the other hand, Hansson et al. showed that after receiving vestibular rehabilitation, stroke patients had improvements in terms of self-rated health such as self-care, mobility, usual activities, pain and discomfort, and depression anxiety measured with EuroQol-5D (EQ5D); however, the difference between the experimental and control groups was not significant [24]. The small sample size and high level of patients' function might be an explanation for no difference between the groups. Moreover, we recommend that vestibular exercises can, to some extent, affect the ADL; however, to be more effective, it is better to consider patients' priorities regarding daily occupations and roles.

## 5. Conclusions

Vestibular rehabilitation was beneficial for fatigue, depression, and ADL in patients with stroke. In addition, the intervention is safe, inexpensive, and easy to implement at the clinic and house using simple tools. Thus, it is recommended for resource-poor and resource-rich societies.

## 6. Study Limitations and Suggestions

One of the limitations of this study was that it was not possible to blind the participants to their treatment status. In addition, the study included only patients who had high levels of cognition as well as functional capabilities and at least 6 months passed since their stroke. Therefore, these results could not be generalized to all stroke patients. All participants in the study were male retired armed personnel with stroke, and women were not included in the survey, which is one of the reasons for the small sample size of the study.

Future works can adopt a longer follow-up phase to achieve more reasonable outcomes to support the effects of vestibular rehabilitation exercises in stroke patients. In addition, considering the associations between sleep, fatigue, and depression in stroke patients [60, 61], the poststroke sleep pattern should also be included as one of the study variables.

## Figures and Tables

**Table 1:** Demographic, clinical characteristics, and group differences of the participants in experimental (*n* = 16) and control (*n* = 16) groups at baseline.

Variables		Experimental group (*n* = 16)	Control group (*n* = 16)	*p* value
Age (years), mean (SD)		58.27 (9.58)	61.61 (7.64)	0.25^∗∗^
Educational level	12 years (*n* (%))	3 (8.3)	2 (5.5)	0.63^∗^
	Above 12 years (*n* (%))	15 (41.6)	16 (44.4)	
Marital status	Single (*n* (%))	3 (8.3)	2 (5.5)	0.63^∗^
	Married (*n* (%))	15 (41.6)	16 (44.4)	
Affected limb	Right (*n* (%))	10 (27.7)	8 (22.2)	0.50^∗^
	Left (*n* (%))	8 (22.2)	10 (27.7)	
Diabetes mellitus	Yes (*n* (%))	6 (16.6)	2 (5.5)	0.10^∗^
	No (*n* (%))	12 (33.3)	16 (44.4)	
Blood pressure	Yes (*n* (%))	7 (19.4)	5 (13.8)	0.48^∗^
	No (*n* (%))	11 (30.5)	13 (36.1)	
Cardiac disease	Yes (*n* (%))	2 (5.5)	7 (19.4)	0.054^∗^
	No (*n* (%))	16 (44.4)	11 (30.5)	
Time since stroke (months), mean (SD)		19.61 (5.05)	19.38 (6.17)	0.67^∗∗∗^
FIS total score mean (SD)		70.38 (13.87)	72.55 (19.84)	0.70^∗∗^
FAS mean (SD)		28.38 (2.78)	29.88 (3.72)	0.18^∗∗^
BDI-II mean (SD)		12.33 (6.56)	12.44 (4.78)	0.95^∗∗^
Barthel Index mean (SD)		85.83 (8.61)	85.00 (8.57)	0.84^∗∗∗^
Lawton ADL mean (SD)		6.50 (1.46)	6.94 (1.43)	0.32^∗∗∗^

^∗^Chi-square test (*α*). ^∗∗^Independent *t*. ^∗∗∗^Mann–Whitney *U*. Abbreviations: FIS = Fatigue Impact Scale; FAS = Fatigue Assessment Scale; BDI-II = Beck Depression Inventory-II.

**Table 2:** The within-group and between-group analyses of the Fatigue Impact Scale, Fatigue Assessment Scale, Beck Depression Inventory-II, Barthel Index, and Lawton ADL Scale in two groups, the experimental group (16 patients received vestibular rehabilitation interventions) and the control group (16 patients received conventional rehabilitation interventions).

Variables		Experimental group		Within group *p*	Control group		Within group *p*	Between groups after treatment		
		Baseline mean (SD)	Outcome mean (SD)		Baseline mean (SD)	Outcome mean (SD)		Mean difference (SE)	*p* value	Effect size
FIS	Physical	25.0 (5.11)	21.16 (6.11)	0.000^∗^	28.11 (8.43)	27.88 (8.50)	0.042^∗^	6.72 (2.46)	0.01^∗∗∗^	0.38
	Cognitive	7.77 (2.23)	6.88 (2.44)	0.000^∗^	7.05 (2.97)	7.05 (2.97)	1^∗^	0.16 (0.90)	0.85^∗∗∗^	—
	Social	37.61 (8.81)	33.72 (9.92)	0.000^∗^	37.38 (9.03)	37.38 (8.99)	1.00^∗^	3.66 (3.15)	0.25^∗∗∗^	—
	Total	70.38 (13.87)	61.77 (16.73)	0.000^∗^	72.55 (19.84)	72.27 (19.83)	0.056^∗^	10.50 (6.11)	0.95^∗∗∗^	—
FAS		28.38 (2.78)	23.16 (3.32)	0.000^∗^	29.88 (3.72)	29.61 (3.77)	0.096^∗^	6.44 (1.18)	0.000^∗∗∗^	1.34
BDI-II		12.33 (6.56)	9.05 (5.59)	0.001^∗^	12.44 (4.78)	12.33 (4.88)	0.163^∗^	3.27 (1.75)	0.070^∗∗∗^	—
BI		85.83 (8.61)	87.77 (7.90)	0.038^∗∗^	85.00 (8.57)	85.27 (8.65)	0.317^∗∗^	-2.50 (2.76)	0.37^∗∗∗^	—
Lawton ADL		6.50 (1.46)	7.66 (1.53)	0.005^∗∗^	6.94 (1.43)	7.05 (1.39)	0.157^∗∗^	-0.61 (0.48)	0.21^∗∗∗^	—

Notes: data were presented as mean (standard deviation) and mean difference (Std. error difference). ^∗^Paired *t*-test. ^∗∗^Wilcoxon signed rank test. ^∗∗∗^Independent *t*. Abbreviations: FIS = Fatigue Impact Scale; FAS = Fatigue Assessment Scale; BDI-II = Beck Depression Inventory-II; BI = Barthel Index; Lawton ADL = Instrumental Activities of Daily Living.

## Data Availability

All data used to support the results of this study are included in the article.

## Appendix

### A. Vestibular Rehabilitation Protocol

#### A.1. Static Position: Standing and Half Kneeling

- Firm surface
- Foam surface
- Trampoline
- Tilt board

Each item was performed with open and closed eyes and head rotations to each side as well as throwing and catching a ball.

#### A.2. Dynamic Position: Walking

- Tandem gait forward and backward
- Walking with a ball in hand and turning side to side as well as tracking the ball
- Stop and start walking, rotating 180 degrees in the direction as well as standing on one leg while it was ordered

#### A.3. Oculomotor Training

- Saccade: rapid eye movement between 2 objects in 4 directions (horizontal, vertical, and 2 diagonal directions)
- Smooth pursuit: tracking an object in 4 directions, while the head is stable
- Vestibuloocular movements: rotating the head side to side, up, and down, while gazing at a subject

## References

[1] Aali G., Drummond A., das Nair R., Shokraneh F. (2020). Post-stroke fatigue: a scoping review. *F1000Research*.

[2] Skogestad I. J., Kirkevold M., Larsson P. (2021). Post-stroke fatigue: an exploratory study with patients and health professionals to develop a patient-reported outcome measure. *Journal of Patient-Reported Outcomes*.

[3] MacIntosh B. J., Edwards J. D., Kang M. (2017). Post-stroke fatigue and depressive symptoms are differentially related to mobility and cognitive performance. *Frontiers in Aging Neuroscience*.

[4] Ponchel A., Bombois S., Bordet R., Hénon H. (2015). Factors associated with poststroke fatigue: a systematic review. *Stroke research and treatment*.

[5] McGeough E., Pollock A., Smith L. N. (2009). Interventions for post-stroke fatigue. *Cochrane Database of Systematic Reviews*.

[6] Acciarresi M., Bogousslavsky J., Paciaroni M. (2014). Post-stroke fatigue: epidemiology, clinical characteristics and treatment. *European Neurology*.

[7] Lan Nguyen Hoang C., Salle J. Y., Mandigout S., Hamonet J., Macian-Montoro F., Daviet J. C. (2012). Physical factors associated with fatigue after stroke: an exploratory study. *Topics in Stroke Rehabilitation*.

[8] Park J. Y., Chun M. H., Kang S. H., Lee J. A., Kim B. R., Shin M. J. (2009). Functional outcome in poststroke patients with or without fatigue. *American Journal of Physical Medicine & Rehabilitation*.

[9] Schepers V. P., Visser-Meily A. M., Ketelaar M., Lindeman E. (2006). Poststroke fatigue: course and its relation to personal and stroke-related factors. *Archives of Physical Medicine and Rehabilitation*.

[10] Gbiri C., Olawale O., Ogunmola O. (2018). Task-oriented circuit training for post-stroke fatigue, sleep-pattern, functional performance and societal re-integration in stroke survivors with post-stroke fatigue: a clinical controlled study. *international journal of stroke*.

[11] Choi-Kwon S., Kim J. S. (2011). Poststroke fatigue: an emerging, critical issue in stroke medicine. *International Journal of Stroke*.

[12] Durand M. J., Boerger T. F., Nguyen J. N. (2019). Two weeks of ischemic conditioning improves walking speed and reduces neuromuscular fatigability in chronic stroke survivors. *Journal of applied physiology (Bethesda, Md. : 1985)*.

[13] Shahar N., Schwartz I., Portnoy S. (2019). Differences in muscle activity and fatigue of the upper limb between task- specific training and robot assisted training among individuals post stroke. *Journal of Biomechanics*.

[14] Alahmari W., Alhowimel A., Alotaibi M., Kontou E., Logan P., Coulson N. (2019). Effectiveness of physiotherapy interventions for post stroke fatigue (PSF): a systematic review. *Physiotherapy*.

[15] Oliveira C. B., Medeiros Í. R. T., Greters M. G. (2011). Abnormal sensory integration affects balance control in hemiparetic patients within the first year after stroke. *Clinics*.

[16] Jang S. H., Lee J.-H. (2016). Impact of sensory integration training on balance among stroke patients: sensory integration training on balance among stroke patients. *Open medicine (Warsaw, Poland)*.

[17] Sharony A. F., Engel-Yeger B. (2021). Sensory modulation and participation in daily occupations in stroke survivors. *Canadian Journal of Occupational Therapy*.

[18] Bernard-Espina J., Beraneck M., Maier M. A., Tagliabue M. (2021). Multisensory integration in stroke patients: a theoretical approach to reinterpret upper-limb proprioceptive deficits and visual compensation. *Frontiers in Neuroscience*.

[19] Han B. I., Song H. S., Kim J. S. (2011). Vestibular rehabilitation therapy: review of indications, mechanisms, and key exercises. *Journal of clinical neurology (Seoul, Korea)*.

[20] Black F. O., Angel C. R., Pesznecker S. C., Gianna C. (2000). Outcome analysis of individualized vestibular rehabilitation protocols. *Otology & Neurotology*.

[21] Tramontano M., Bergamini E., Iosa M., Belluscio V., Vannozzi G., Morone G. (2018). Vestibular rehabilitation training in patients with subacute stroke: a preliminary randomized controlled trial. *NeuroRehabilitation*.

[22] Mitsutake T., Imura T., Tanaka R. (2020). The effects of vestibular rehabilitation on gait performance in patients with stroke: a systematic review of randomized controlled trials. *Journal of Stroke and Cerebrovascular Diseases*.

[23] Mitsutake T., Sakamoto M., Ueta K., Oka S., Horikawa E. (2017). Effects of vestibular rehabilitation on gait performance in poststroke patients: a pilot randomized controlled trial. *International Journal of Rehabilitation Research*.

[24] Ekvall Hansson E., Pessah-Rasmussen H., Bring A., Vahlberg B., Persson L. (2020). Vestibular rehabilitation for persons with stroke and concomitant dizziness—a pilot study. *Pilot and feasibility studies*.

[25] Lopez C. (2016). The vestibular system: balancing more than just the body. *Current Opinion in Neurology*.

[26] Angelaki D. E., Cullen K. E. (2008). Vestibular system: the many facets of a multimodal sense. *Annual Review of Neuroscience*.

[27] Sarvghadi P., Ghaffari A., Rostami H. R. (2019). The effects of neurofeedback training on short-term memory and quality of life in women with breast cancer. *International Journal of Therapy and Rehabilitation*.

[28] Oppenländer K., Keller I., Karbach J., Schindler I., Kerkhoff G., Reinhart S. (2015). Subliminal galvanic-vestibular stimulation influences ego- and object-centred components of visual neglect. *Neuropsychologia*.

[29] Angelaki D. E., Klier E. M., Snyder L. H. (2009). A vestibular sensation: probabilistic approaches to spatial perception. *Neuron*.

[30] Cumming T. B., Mead G. (2017). Classifying post-stroke fatigue: optimal cut-off on the fatigue assessment scale. *Journal of Psychosomatic Research*.

[31] Ansari N. N., Naghdi S., Hasson S., Valizadeh L., Jalaie S. (2010). Validation of a mini-mental state examination (MMSE) for the Persian population: a pilot study. *Applied Neuropsychology*.

[32] Frith J., Newton J. (2010). Fatigue impact scale. *Occupational Medicine*.

[33] Fisk J. D., Ritvo P. G., Ross L., Haase D. A., Marrie T. J., Schlech W. F. (1994). Measuring the functional impact of fatigue: initial validation of the fatigue impact scale. *Clinical Infectious Diseases*.

[34] Saneii S. H., Heidari M., Zaree M., Akbarfahimi M. (2020). Psychometric features of the Persian version of the fatigue impact scale in Iranian stroke patients. *Journal of Advances in Medical and Biomedical Research*.

[35] Shahid A., Wilkinson K., Marcu S., Shapiro C. M. (2011). Fatigue assessment scale (FAS). *STOP, THAT and One Hundred Other Sleep Scales*.

[36] Lookzadeh S., Kiani A., Taghavi K. (2018). Evaluation of the reliability and validity of the Persian version of the fatigue assessment scale in Iranian sarcoidosis patients. *Open Access Macedonian Journal of Medical Sciences*.

[37] Cheraghifard M., Sarlak N., Taghizadeh G., Azad A., Fallah S., Akbarfahimi M. (2022). Minimal and robust clinically important difference of three fatigue measures in chronic stroke survivors. *Topics in Stroke Rehabilitation*.

[38] Brantley P. J., Dutton G. R., Wood K. B. (2004). *The Beck Depression Inventory-II (BDI-II) and the Beck Depression Inventory-Primary Care (BDI-PC)*.

[39] Ghassemzadeh H., Mojtabai R., Karamghadiri N., Ebrahimkhani N. (2005). Psychometric properties of a Persian-language version of the Beck Depression Inventory - second edition: BDI-II-Persian. *Depression and Anxiety*.

[40] Mahoney F. I., Barthel D. W. (1965). Functional evaluation: the Barthel Index. *Maryland State Medical Journal*.

[41] Oveisgharan S., Shirani S., Ghorbani A. (2006). Barthel Index in a Middle-East country: translation, validity and reliability. *Cerebrovascular Diseases*.

[42] Graf C. (2008). The Lawton instrumental activities of daily living scale. *AJN The American Journal of Nursing*.

[43] Mehraban A. H., Soltanmohamadi Y., Akbarfahimi M., Taghizadeh G. (2014). Validity and reliability of the Persian version of Lawton instrumental activities of daily living scale in patients with dementia. *Medical Journal of the Islamic Republic of Iran*.

[44] Cass S. P., Borello-France D., Furman J. M. (1996). Functional outcome of vestibular rehabilitation in patients with abnormal sensory-organization testing. *The American Journal of Otology*.

[45] Gittler M., Davis A. M. (2018). Guidelines for adult stroke rehabilitation and recovery. *JAMA*.

[46] Cumming G. (2013). Cohen’sd needs to be readily interpretable: comment on Shieh (2013). *Behavior Research Methods*.

[47] Abasi A., Raji P., Friedman J. H. (2020). Effects of vestibular rehabilitation on fatigue and activities of daily living in people with Parkinson’s disease: a pilot randomized controlled trial study. *Parkinson’s Disease*.

[48] Hebert J. R., Corboy J. R., Manago M. M., Schenkman M. (2011). Effects of vestibular rehabilitation on multiple sclerosis–related fatigue and upright postural control: a randomized controlled trial. *Physical Therapy*.

[49] Sun Y., Zehr E. P. (2019). Training-induced neural plasticity and strength are amplified after stroke. *Exercise and Sport Sciences Reviews*.

[50] Fathi S., Taghizadeh G., Azad A. (2021). Effects of upper extremity coordination exercises based on fatigue prediction on upper extremity sensory-motor functions in chronic stroke survivors. *Iranian-Rehabilitation-Journal*.

[51] Bajaj S., Butler A. J., Drake D., Dhamala M. (2015). Brain effective connectivity during motor-imagery and execution following stroke and rehabilitation. *NeuroImage: Clinical*.

[52] Amengual J. L., Münte T. F., Marco-Pallarés J. (2014). Overactivation of the supplementary motor area in chronic stroke patients. *Journal of Neurophysiology*.

[53] Galligan N. G., Hevey D., Coen R. F., Harbison J. A. (2016). Clarifying the associations between anxiety, depression and fatigue following stroke. *Journal of Health Psychology*.

[54] Lerdal A., Bakken L. N., Rasmussen E. F. (2011). Physical impairment, depressive symptoms and pre-stroke fatigue are related to fatigue in the acute phase after stroke. *Disability and Rehabilitation*.

[55] Ghaffari A., Akbarfahimi M., Rostami H. R. (2020). Discriminative factors for post-stroke depression. *Asian Journal of Psychiatry*.

[56] Llorca G. E., Castilla-Guerra L., Moreno M. F., Doblado S. R., Hernández M. J. (2015). Depresion post ictus: una actualizacion. *Neurología (English Edition)*.

[57] Ghaffari A., Rostami H. R., Akbarfahimi M. (2021). Predictors of instrumental activities of daily living performance in patients with stroke. *Occupational Therapy International*.

[58] Maclean N., Pound P., Wolfe C., Rudd A. (2000). Qualitative analysis of stroke patients’ motivation for rehabilitation. *BMJ*.

[59] Dai C. Y., Huang Y. H., Chou L. W., Wu S. C., Wang R. Y., Lin L. C. (2013). Effects of primary caregiver participation in vestibular rehabilitation for unilateral neglect patients with right hemispheric stroke: a randomized controlled trial. *Neuropsychiatric Disease and Treatment*.

[60] Ho L. Y. W., Lai C. K. Y., Ng S. S. M. (2021). Contribution of sleep quality to fatigue following a stroke: a cross-sectional study. *BMC Neurology*.

[61] Dong L., Brown D. L., Chervin R. D., Case E., Morgenstern L. B., Lisabeth L. D. (2021). Pre-stroke sleep duration and post-stroke depression. *Sleep Medicine*.

